# Supplementary material for: Influence of geographic access and socioeconomic characteristics on breast cancer outcomes: A systematic review
Source: PLoS One. 2022 Jul 19;17(7):e0271319. doi: 10.1371/journal.pone.0271319 (PMC9295987; doi:10.1371/journal.pone.0271319)
Supplement: S1 File — (PDF) [file pone.0271319.s002.pdf]

Quality assessment results using an adaptive Effective Public Health Practice Project (EPHPP) tool

AUTHOR (YEAR)	DOMAINS					OVERALL SCORE
	STUDY DESIGN	SELECTION BIAS	CONFOUNDERS*	DATA COLLECTION	DATA ANALYSES	
Baade et al., 2016	2	1	2	1	1	1
Celaya et al., 2010	2	1	1	2	1	1
Dai, 2010	2	1	2	2	1	1
Dasgupta et al., 2016	2	2	1	2	2	2
Dasgupta et al., 2017	2	1	1	2	1	1
Engelman et al., 2002	2	2	2	1	1	1
Goovaerts, 2010	2	3	2	3	1	3
Henry et al., 2013	2	1	1	1	1	1
Henry et al., 2011	2	1	1	1	1	1
Henry et al., 2014	2	2	1	2	1	1
Huang et al., 2009	2	2	1	2	1	1
Jones et al., 2008	2	2	2	2	1	2
Kim et al., 2013	2	1	1	1	1	1
Lian et al., 2012	2	1	2	2	1	1
Lin et al., 2018	2	1	2	2	1	1
Lin and Wimberly, 2017	2	1	2	2	1	1
McLafferty et al., 2011	2	2	2	2	1	2
Onitilo et al., 2013	2	3	3	2	1	3
Rocha-Brischiliari et al., 2018	2	3	2	2	2	2
Sauerzapf et al., 2008	2	2	2	2	1	2
Schroen and Lohr, 2009	2	2	2	3	2	2
St-Jacques et al., 2013	2	1	2	1	1	1
Tarlov et al., 2009	2	1	2	1	1	1
Voti et al., 2006	2	1	1	1	1	1
Yang and Wapnir, 2018	2	3	1	1	1	2

*Relevant confounders: age, partner status, educational level, health assurance status, ethnicity, SES at contextual level;
Quality assessment rating: 1 (strong), 2 (moderate), 3 (weak)
