# Supplementary material for: Influence of geographic access and socioeconomic characteristics on breast cancer outcomes: A systematic review
Source: PLoS One. 2022 Jul 19;17(7):e0271319. doi: 10.1371/journal.pone.0271319 (PMC9295987; doi:10.1371/journal.pone.0271319)
Supplement: Attachment — Submitted filename: Response to Reviewers.docx [file pone.0271319.s004.docx]

From:

Benoit Conti (corresponding author)

Laboratoire Ville, Mobilité, Transport

Université Gustave Eiffel

benoit.conti@univ-eiffel.fr

Manuscript Number: PONE-D-21-10021R1

To:

Editorial board,

Plos One

Paris, France, 14th February 2022

Dear Editor-in-Chief,

Dear Tzai-Hung Wen,

We would like to thank you for accepting the attached original revised manuscript entitled “Influence of geographic access and socioeconomic characteristics on breast cancer outcomes: a systematic review” for your consideration towards publication in *Plos One*.

In response to the previous review, we have carefully examined the comments made by the second reviewer. All corrections and explanations required have been added to the revised manuscript. We feel that we have answered the reviewer’s questions, commented on the remarks point by point in the “responses to reviewers” document and modified our paper accordingly. All modifications appear in the manuscript highlighted in yellow and are included in the revised manuscript.

The word count for the main text is now 5,672 without references. The paper includes 3 tables and 7 figures in the main text. All correspondence may be directed to Benoit Conti at the address below. We would be delighted to provide any further information that may be required. We hope you will consider this paper suitable for publication in your journal. We look forward to receiving your editorial decision.

Yours sincerely,

Benoit Conti, on behalf of the authors

Manuscript Number: PONE-D-21-10021

**REVISION**

Influence of geographic access and socioeconomic characteristics on breast cancer outcomes: a systematic review

**We thank the editor and the reviewers for their positive comments on our paper. We have addressed the concerns that have been raised and we provide below a point-by-point reply to the comments of the two reviewers.**

Reviewer #1

Thanks for addressing my previous comments. The current version looks good to me. I don't have further questions.

**Reply: We thank the reviewer for his/her appreciation of our review paper and his/her previous suggestions for improvement.**

Reviewer #2:

**We thank the reviewer for his/her appreciation of our review paper and his/her suggestions for improvement. We have taken into account all the comments of the reviewer and provide below our point-by-point answers.**

1. In the introduction part, the authors propose two questions in this study. First, in the context of equal geographic access to healthcare facilities, do women with disadvantaged social and economic characteristics have poorer breast cancer outcomes than more advantaged women? Second, in the case of equal socioeconomic level, do women with poor geographic access to healthcare facilities have worse breast cancer outcomes than women with higher geographic access? However, the authors present the results by 4 parts which included: What measures of breast cancer outcomes, geographic access, and SES characteristics? What are the relationships between geographic access to health-care facilities and breast cancer outcomes? What are the relationships between SES characteristics and breast cancer outcomes? What are the combined effects of geographic access and SES characteristics on breast cancer outcomes? I suggest the questions which are proposed in introduction should include those four questions to match the structure of results.

**Reply: As recommended by the reviewer, we have modified the questions which are proposed in introduction and included the four questions to match the structure of results (Introduction section (L85 to 90)). The end of the introduction now reads as follows: *To answer these two general questions, the result section will be divided into four research questions: (i) what measures of breast cancer outcomes, geographic access, and SES characteristics? (ii) What are the relationships between geographic access to health-care facilities and breast cancer outcomes? (iii) What are the relationships between SES characteristics and breast cancer outcomes? (iv) What are the combined effects of geographic access and SES characteristics on breast cancer outcomes?***

2. If the study brings the thought on taking account of the fact that people do not necessarily use the closest facility, and that they do not necessarily start from home. I suggest the authors can give some advice on the methods or measurements to the future studies. How should we take account those facts into our researches?

**Reply: We thank the reviewer for his/her comments. A sentence already present in the article offers an initial response to your comment in the discussion section (L360-364). As recommended by the reviewer we add a sentence on this methodological issue (L364-366):** ***To this purpose, information on where women come from when they go to hospital and why they choose (or not choose) their hospital should be included both in questionnaire and interview.***

3. The same issue as the second comment. If women who live in suburban or rural areas are more willing to travel longer distances than women living in urban centers. How can we design our method to explore this possibility or realty? The authors can give the readers some advices on some arguments which are this study discussed.

**Reply: We thank the reviewer for his/her comments. As noted by the reviewer, considering the local residential context is needed to better understand travel distance to healthcare. One option could be to systematically characterize this local context with information from self-reported women (questionnaire) or/and from spatial databases on urban density (e.g. urban, suburban, rural). In this way, we add a sentence on this methodological issue (L409-411): *To this purpose, information on urban density level of residential area of women should be collected either from self-reported questionnaires or from spatial databases (e.g., urban, suburban, rural areas).***

4. The conclusion part has already mentioned some directions for the future studies. However, I strongly suggest the research team can give the readers all the implications which can response to all interesting results from your analysis.

**Reply: We thank the reviewer for his/her comments. As recommended by the reviewer we add a sentence about the implications in the conclusion section: *This requires a specific research protocol based on regular questionnaires and/or interviews at different times, from diagnosis to one or more years later, including dimensions of precariousness and ability to travel as well as spatial access to healthcare (e.g. address of their general practitioners (GP), possession of a driving licence, availability of public transport).***
